# PileLine: a toolbox to handle genome position information in next-generation sequencing studies

**DOI:** 10.1186/1471-2105-12-31

**Published:** 2011-01-24

**Authors:** Daniel Glez-Peña, Gonzalo Gómez-López, Miguel Reboiro-Jato, Florentino Fdez-Riverola, David G Pisano

**Affiliations:** 1 Higher Technical School of Computer Engineering, University of Vigo, Ourense, Spain; 2 Bioinformatics Unit (UBio), Structural Biology and Biocomputing Programme, Spanish National Cancer Research Centre (CNIO), Madrid, Spain

## Abstract

**Background:**

Genomic position (GP) files currently used in next-generation sequencing (NGS) studies are always difficult to manipulate due to their huge size and the lack of appropriate tools to properly manage them. The structure of these flat files is based on representing one line per position that has been covered by at least one aligned read, imposing significant restrictions from a computational performance perspective.

**Results:**

PileLine implements a flexible command-line toolkit providing specific support to the management, filtering, comparison and annotation of GP files produced by NGS experiments. PileLine tools are coded in Java and run on both UNIX (Linux, Mac OS) and Windows platforms. The set of tools comprising PileLine are designed to be memory efficient by performing fast seek on-disk operations over sorted GP files.

**Conclusions:**

Our novel toolbox has been extensively tested taking into consideration performance issues. It is publicly available at http://sourceforge.net/projects/pilelinetools under the GNU LGPL license. Full documentation including common use cases and guided analysis workflows is available at http://sing.ei.uvigo.es/pileline.

## Background

Nowadays, commercially available NGS technologies are able to generate in a single run millions of DNA short reads producing a gigabasepair (Gbp) scale throughput at relatively low cost [1]. A crucial step in NGS resequencing workflows is the mapping of DNA short reads against a reference genome in order to obtain text files which represent the sequence information by its genomic position (e.g. *.pileup*, *.bed*, *.gff*, *.vcf*, etc.). In this context, SAMtools [2] is a well-known open-source package able to manipulate Sequence Alignment Map (SAM) files and, amongst other utilities, its variant caller generates a GP file called pileup able to be further analyzed in NGS-based research studies (e.g. DNA or RNA-seq).

As an example, Ding et al. [3] have characterized novel mutations using data analysis workflows that require a comprehensive GP file handling for variant annotation (i.e. to discard known variants such as SNPs) and case-control comparisons. Nevertheless, the management of this type of files is usually cumbersome and inefficient due to both their large size (about 3·10^9 ^lines for human genome) and the lack of specific tools able to efficiently manage them from a disk, memory and CPU point of view.

Starting from our experience in giving direct support to wet-lab users requiring NGS analysis we have developed PileLine, a novel and flexible command-line toolbox for efficient management, filtering, comparison and annotation of GP files. The toolbox has been designed to be memory efficient by performing fast seek on-disk operations over sorted GP files. Based on the combination of basic core operations, PileLine provides several functionalities, including (*i*) full standard annotation with human dbSNP, HGNC Gene Symbol and Ensembl IDs, (*ii*) custom annotation through standard *.bed *or *.gff *files, (*iii*) two sample (i.e.: case VS control) and *n *sample comparison at variant level, (*iv*) generation of SIFT [4], Firestar [5] and PolyPhen [6] compatible outputs for predicting the consequences of non-synonymous coding variants on protein function, and (*v*) a genotyping quality control (QC) test for estimating performance metrics on detecting homo/heterozygote variants against a given gold standard genotype [7].

## Implementation

PileLine is coded in Java and consists of a set of command-line utilities that are easy to integrate in custom workflows or user-friendly frameworks like Galaxy [8]. The tools comprising PileLine are focussed on two different but complementary activities: (*i*) *processing and annotation*, implementing simple but reusable operations over input GP files and (*ii*) *analysis*, giving support to more advanced and specific requirements (see Table 1 and Additional File 1).

**Table 1:** Summary of PileLine functionalities

Tool	Description
*Processing and annotation*	

fastseek	Retrieves all lines within a specified genome range.
fastjoin	Joins two GP input files by genomic coordinate. It can also perform left- and right- outer joins which print orphan lines.
rfilter	Selects only those positions inside at least one of a given set of intervals (*.bed *or *.gff *files). It also implements an annotation mode to report all positions plus an extra column containing all the intervals in which each position is contained.
sort	Sorts a GP file by genomic coordinate. SAMtools generated pileup files are usually sorted.
pileup2sift	Generates a SIFT-compatible change column for each variant line in the GP file.
pileup2polyphen	Generates a Polyphen2-compatible change column for each variant line in the GP file.
pileup2firestar	Generates a firestar-compatible input for each variant line in the GP file.

*Analysis*	

2smc	Compares two samples (i.e. case VS control) by retrieving all positions where the genotype is discrepant between the two samples. For each sample a variant GP file is needed, as well as the complete GP file (which includes the invariant positions).
nsmc	Compares *n *samples of two conditions (i.e. case VS control). Taking one GP file per sample, it reports those samples containing each position and also performs a Fisher's exact test to find reproducible and characteristic positions.
genotest	Performs a QC test on genotyping. Compares two genotypes (experimental VS gold standard) and evaluates the performance on detecting homo/heterozygous variants. It also generates data to plot a ROC curve in order to estimate the best SNP quality threshold. See Additional File 1 for an example output.

**Figure 1:**
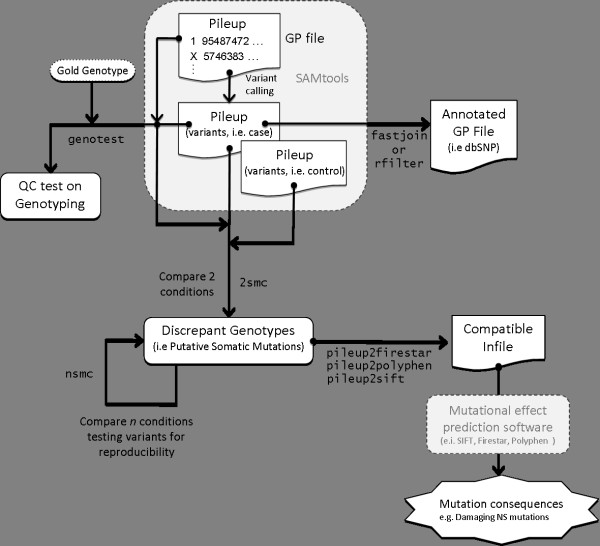
**An example of a PileLine workflow working with SAMTools**. *.pileup *files generated by SAMtools are processed by PileLine in order to perform QC tests, annotation and sample comparison (NS: non-synonymous).

The primary input data of PileLine are GP files (e.g. *.pileup *from SAMtools) containing the chromosome name and the coordinate position as the two first columns (see Figure 1). The main design principle of the PileLine toolbox is to avoid loading input data into memory, so core functions operate directly on disk. One of the available command-line tools is *fastseek*, which performs a direct binary search on sorted GP files without requiring an additional index to be created. This functionality provides direct access to any range of genomic coordinates without loading the whole file into memory. Initially, *fastseek *finds the first and last lines of each sequence and next, performs a binary search on the lines belonging to the queried sequence in order to find the first position within the specified range.

The second design principle of PileLine is focussed on flexibility and modularity. Thus, PileLine tools may be combined with standard UNIX commands allowing custom data analysis workflows. Moreover, the modular design of our toolbox facilitates the inclusion of additional functionalities. With respect to the file formats, while *2smc*, *nsmc*, *pileup2sift*, *pileup2polyphen *and *pileup2firestar *work with specific samtools .pileup file format, *fastseek*, *fastjoin*, *rfilter*, *sort *and *genotest *work with generic GP files (i.e.: .pileup, .vcf, .gff, .bed, etc.).

## Results and discussion

PipeLine toolbox contains 10 command-line utilities that have been designed to be memory efficient by performing on-disk operations over sorted GP files. By combining their execution using different arguments and several options the user is able to sketch and execute diverse workflows that can be enhanced by using third party software applications. Here we report several example PileLine applications, further commands and examples of use can be found on the PileLine web site:

a) Case-Control comparisons working with *.pileup *files:

$ sh YOUR_PATH_TO_PILELINE/cmd/**pileline-2smc.sh **-a <Control1.pileup> -b <Case1.pileup> -v <Control1.variants.pileup> -w <Case1.variants.pileup> -o <myoutput1.txt>

b) *N *sample comparisons reporting consistent variants amongst *.pileup *files:

$ sh YOUR_PATH_TO_PILELINE/cmd/**pileline-nsmc.sh **--a-samples <Control1.variants.pileup>,<Control2.variants.pileup>,<Control3.variants.pileup> --b-samples <Case1.variants.pileup>,<Case2.variants.pileup>,<Case3.variants.pileup> -o <mycommonvariants_in_Cases.txt>

c) Full annotation of GP files with human dbSNP:

$ sh YOUR_PATH_TO_PILELINE/cmd/**pileline-fastjoin.sh **-a <GP_file.txt> -b ./dbSNP_36.3.txt --left-outer-join ><mydbSNPannotation1.txt>

#HGNC Gene Symbol, Ensembl IDs and custom annotations are also allowed and may be supplied through standard *.bed *or *.gff *files.

d) Generate input for external mutational effects prediction software (i.e. SIFT):

$ sh YOUR_PATH_TO_PILELINE/cmd/**pileline-pileup2sift**.**sh **-i <myfile.pileup> > <mysiftinput.txt>

# Polyphen2 and Firestar inputs are also allowed.

e) Print a given range of a GP file (without indexing).

$ sh YOUR_PATH_TO_PILELINE/cmd/**pileline-fastseek.sh **-p <GP_file> -s chr10:100:10000 ><mychrrange.txt>

PileLine performs efficiently on a standard PC (Intel Core 2,33 GHz with 1 Gb RAM), where initial tests with the *fastseek *command showed good performance being able to retrieve 1400 random positions per second on a file of ~174 millions of lines (~5,5 Gb). This behaviour allows, for example, to retrieve all positions from a *.pileup *file containing known SNPs (~4 million) in approximately 45 minutes consuming less than 150 Mb of RAM.

Although an optimal search performance could be attained by using auxiliary indexes, this approach requires an additional step for building the supporting files. Moreover, the performance degrades linearly as the input GP file grows in size, and its generation takes a considerable amount of time. PileLine was designed to avoid indexing but, by performing binary searches instead of sequential searches (taking advantage of sorted GP files), it scales reasonably well since its complexity is O(log_2_N), where N is the number of lines of the input GP file.

The PileLine toolbox is maintained and distributed using a concurrent CVS version control system at SourceForge. The community platform provides wiki support through MediaWiki.

## Conclusion

In this work we have presented PileLine, a toolbox for the efficient processing of standard genomic position files such as *.pileup*, *.bed*, *.gff *and *.vcf*. Based on the combination of basic core tools, PileLine provides a catalogue of functions to analyze, compare, filter and annotate GP files giving support to common NGS analysis workflows. Given the growing number of NGS-oriented applications, PileLine has been implemented using a modular design to facilitate the inclusion of new functionalities. PileLine toolbox and source code are freely available from http://sourceforge.net/projects/pilelinetools and licensed under the terms of the GNU Lesser General Public License. Examples of use, commands help, guided analysis workflows, example files and full documentation are available in PileLine's wiki at http://sing.ei.uvigo.es/pileline.

## Availability and requirements

**Project name**: PileLine

**Project home page**: http://sing.ei.uvigo.es/pileline

**Operating systems**: Windows, Unix-like (Linux, Mac OSX)

**Programming language**: Java

**Other requirements**: Java Runtime Environment (JRE) 1.6, Apache Ant 1.7

**License**: GNU LGPL

## Authors' contributions

DG-P programmed the PileLine application. GGL provided use cases, tested the usability of the software and generated PileLine documentation. MRJ tested the performance of PileLine tools. DG-P and GGL wrote the paper while FFR and DGP provided comments and discussion. All authors read and approved the final manuscript.

## Supplementary Material

Additional file 1 **Example output of a genotyping test for quality control**. Genotest metrics table description. It may be obtained by using --print-help-table argument. Click here for file
