# Supplementary material for: PileLine: a toolbox to handle genome position information in next-generation sequencing studies
Source: BMC Bioinformatics. 2011 Jan 24;12:31. doi: 10.1186/1471-2105-12-31 (PMC3037855; doi:10.1186/1471-2105-12-31)
Supplement: Additional file 1 — Example output of a genotyping test for quality control. Genotest metrics table description. It may be obtained by using --print-help-table argument. [file 1471-2105-12-31-S1.PDF]

###Table description

	REAL			
CANDIDATE	HETEROZYGOUS	HOMO. ALTER	HOMO. REF	NN
HETEROZYGOUS	A1*	A2	A3	A4
HOMO. ALTER	B1	B2**	B3	B4
HOMO. REF	C1	C2	C3	C4
NN	D1	D2	D3	D4

Coverage Rate: 1 - (D1+D2+D3)/(A1+A2+A3+B1+B2+B3+C1+C2+C3+D1+D2+D3)

Senquencing accuracy: (A1+B2+C3)/(A1+A2+A3+B1+B2+B3+C1+C2+C3)
Variant accuracy: (A1+B2)/(A1+A2+B1+B2+C1+C2)
Sensitivity (recall): (A1+A2+B1+B2)/(A1+A2+B1+B2+C1+C2)
Precision (PPV):(A1+A2+B1+B2)/(A1+A2+A3+B1+B2+B3)
Specificity:(C3)/(A3+B3+C3)
F1-score: 2*((recall*precision)/(recall+precision))
kappa: (kappa_hits_obs - kappa_hits_expected)/(1 - kappa_hits_expected),
where:
kappa_no_samples: A1+A2+A3+B1+B2+B3+C1+C2+C3
kappa_hits_obs: (A1+B2+C3)/kappa_no_samples
kappa_hits_expected: (((A1+B1+C1)/kappa_no_samples)*((A1+A2+A3)/kappa_no_samples))+(((A2+B2+C2)/kappa_no_samples)*((B1+B2+B3)/kappa_no_samples))+(((A3+B3+C3)/kappa_no_samples)*((C1+C2+C3)/kappa_no_samples))
Youden's J: sensitivity + specificity - 1
fpr:(A3+B3)/(A3+B3+C3)
fdr:(A3+B3)/(A1+A2+A3+B1+B2+B3)
fnr:(C1+C2)/(A1+A2+B1+B2+C1+C2)
Variant discrepancy rate: (A2+B1)/(A1+A2+B1+B2)
* Errors: Both heterozygous, but different
** Errors: Both homozygous, but different
